# Parámetros de variabilidad glucémica de la monitorización continua de glucosa como predictores de diabetes: evaluación prospectiva en una población general sin diabetes

**DOI:** 10.1515/almed-2024-0125

**Published:** 2025-01-13

**Authors:** Andrea Valle Rodríguez, Javier Rodríguez García, Felix Camiña Darriba, Juan B. Ortolá Devesa, Santiago Rodríguez-Segade Villamarín

**Affiliations:** Laboratorio de Bioquímica Clínica del Complejo Hospitalario, Universitario de Santiago de Compostela, Santiago de Compostela, España; Departamento de Bioquímica y Biología Molecular, Universidad de Santiago de Compostela, Santiago de Compostela, España

**Keywords:** monitorización continua de glucosa, diabetes, variabilidad glucémica, HbA_1c_, amplitud media de las excursiones glucémicas, desviación estándar

## Abstract

**Objetivos:**

Evaluar prospectivamente la capacidad de distintas métricas de variabilidad glucémica obtenidas mediante monitorización continua de glucosa (MCG) para la predicción del desarrollo de diabetes en una población sin diabetes.

**Métodos:**

Se incluyeron 497 participantes sin diabetes del estudio AEGIS. Los participantes utilizaron un sistema de MCG (iPro2^®^) durante seis días. Se evaluaron las siguientes métricas: desviación estándar (SD), coeficiente de variación (CV) y amplitud media de las excursiones glucémicas (MAGE). Los sujetos fueron seguidos durante una media de 6 años. Se utilizaron curvas ROC para determinar la capacidad predictiva de las métricas de variabilidad glucémica y se calcularon la sensibilidad y especificidad.

**Resultados:**

De los 497 participantes, 16 mujeres (4,9 %) y 9 hombres (5,2 %) desarrollaron diabetes. Las concentraciones iniciales de HbA_1c_ y glucosa en ayunas fueron significativamente más altos en aquellos que progresaron a diabetes. Las métricas de variabilidad glucémica también fueron significativamente mayores en estos individuos (SD: 18 vs. 13 mg/dL; CV: 17 % vs. 14 %; MAGE: 36 vs. 27 mg/dL; p<0,001 en todos los casos). La SD mostró la mayor AUC (0,81), con una sensibilidad del 80 % y una especificidad del 72 % para un punto de corte de 14,9 mg/dL. Las AUC fueron mayores en hombres para todas las métricas estudiadas.

**Conclusiones:**

Las métricas obtenidas por MCG, especialmente la SD, son predictores efectivos de la progresión a diabetes tipo 2 en una población sin diabetes. Estos hallazgos sugieren la utilidad de la variabilidad glucémica en la identificación temprana de individuos en riesgo de desarrollar diabetes.

## Introducción

Los sistemas de monitorización continua de glucosa (MCG) son dispositivos de pequeño tamaño que, mediante un sensor subcutáneo, proporcionan información detallada sobre el comportamiento glucémico. Esta tecnología permite conocer con mayor precisión la magnitud y duración de las oscilaciones glucémicas en comparación con los métodos de medición convencionales [1], 2]. Aunque la MCG es muy útil para el control y seguimiento de las personas con diabetes, su implementación presenta desafíos para los profesionales de la salud, especialmente en cuanto a la gestión de los datos obtenidos y su aplicación clínica. En los últimos años, medidas relacionadas con la MCG, como la variabilidad glucémica y el tiempo en rango, se han integrado en la práctica clínica habitual [3]. En 2019, un comité de expertos en tecnologías de MCG (médicos, investigadores y personas con diabetes) publicó una guía de consenso actualizada para promover el uso correcto y estandarizado de la métrica del tiempo en rango en la práctica clínica, y una revisión más reciente estableció el tiempo en rango como una medida gold-standard [4], 5]. Además, el tiempo en rango se ha reconocido como predictor de complicaciones diabéticas [6].

Diversos estudios realizados en personas con diabetes, tanto tratadas como no tratadas con insulina, han demostrado los beneficios de la MCG en la reducción de peso, la modificación de la dieta y/o el incremento de la actividad física [7], [8], [9], [10], [11], [12], [13]. Sin embargo, actualmente la MCG solo está indicada para personas con diabetes.

Los métodos actuales para diagnosticar la prediabetes proporcionan una “instantánea” del estado glucémico de un individuo, lo cual puede ser engañoso y/o ineficaz para detectar, controlar y gestionar la disglicemia a tiempo. Por ejemplo, la determinación de la HbA_1c_ puede estar falsamente elevada o disminuida en individuos con hemoglobinopatías, enfermedad renal crónica, anemia y otros factores de interferencia [14], [15], [16]. Además, los resultados de las pruebas pueden verse afectados por diferencias étnicas/raciales en las tasas de glicosilación y en situaciones como el embarazo [17], [18], [19], [20]. Los valores de HbA_1c_ representan solo un promedio de las concentraciones de glucosa durante un periodo de 2–3 meses, lo cual impide identificar las afecciones específicas o los comportamientos del paciente que contribuyen a la disglicemia, información esencial para un control glucémico eficaz.

El uso de la MCG en individuos en riesgo (p. ej., sobrepeso/obesidad, antecedentes familiares de T2D) puede superar las limitaciones de los marcadores actuales, ya que proporciona información significativa en formatos que permiten a los clínicos y pacientes identificar fácilmente los patrones glucémicos, así como las fluctuaciones interdía e intradía de la glucosa, que pueden indicar la presencia y/o la gravedad de la disglicemia. Nuestros resultados previos en sujetos sin diabetes muestran que aquellos con valores normales de HbA_1c_ y glucosa en ayunas (FG), pero que a través de la MCG pasan un gran porcentaje del tiempo con concentraciones de glucemia en rango de prediabetes y/o diabetes, también presentan variabilidades glucémicas (VG) superiores a las de otros individuos normoglucémicos, siendo similares a las de los sujetos clasificados como prediabéticos según criterios convencionales [21].

El objetivo principal de este estudio es evaluar de forma prospectiva si, en una amplia muestra de sujetos representativos de la población no diabética, la utilización de tres métricas de variabilidad glucémica determinadas inicialmente por MCG, como el coeficiente de variación (CV), la desviación estándar (SD) y la amplitud media de las excursiones glucémicas (MAGE), *que es una forma de medir la variabilidad glucémica al calcular la amplitud promedio de las fluctuaciones de glucosa que superan una desviación estándar en el MCG,* se asocian con una mayor progresión a diabetes tipo 2.

## Materiales y métodos

### Participantes y diseño del estudio

Los sujetos que forman parte de este estudio son un subconjunto de los 1516 participantes del Estudio de Glicación e Inflamación de A Estrada (AEGIS; ensayo NCT01796184 en www.clinicaltrials.gov) [22], iniciado en 2012. Este es un estudio epidemiológico prospectivo basado en la población, enfocado en las relaciones entre diversas pruebas de disglicemia e inflamación y el riesgo de progresión a diabetes y enfermedad cardiovascular.

De los 1516 participantes, se consideró que 1065 cumplían los requisitos básicos para la MCG (capacidad para seguir el protocolo y abstenerse de comer fuera de casa, y ausencia de alergia a los adhesivos o cualquier otra afección médica que pudiera afectar los datos obtenidos mediante la MCG). De estos 1065, 622 aceptaron participar y completaron un periodo de 6 días de MCG. Este trabajo se centra en el subconjunto de 497 miembros de estos 622 que cumplían los siguientes requisitos adicionales [1]: estar clínicamente estables, sin enfermedades agudas ni antecedentes de diátesis o enfermedades renales o hepáticas crónicas [2]; tener una glucosa en ayunas<126 mg/dL y una HbA_1c_ < 6,5 % [48 mmol/mol], tanto cuando se instaló el sensor de MCG como cuando se retiró una semana después [3]; no tomar fármacos que puedan afectar al metabolismo de la glucosa durante el periodo de MCG [4]; no estar embarazadas; y [5] se excluyeron los sujetos con registros de MCG incompletos (<2 días completos). “*El diagnóstico de diabetes se definió de acuerdo con el criterio de la Asociación Americana de Diabetes [23]*.”

### Monitorización continua de glucosa [MCG]

Previamente se ha descrito un análisis detallado de la MCG [21]. Brevemente, el día 0, una enfermera especializada insertó el sistema de MCG (iPro2^®^ de Medtronic Minimed, Northridge, CA) en cada uno de los participantes seleccionados, después de que estos completarán un ayuno nocturno. Al cabo de una hora, se tomaron muestras de sangre para determinar la glucosa en ayunas y la HbA_1c_. Los participantes recibieron instrucciones sobre el cuidado y la calibración del dispositivo MCG, y se les pidió que lo llevaran durante siete días consecutivos sin alterar sus hábitos dietéticos o de actividad. Para la calibración, se les enseñó a utilizar un medidor de glucosa que ofrece valores equivalentes en plasma (OneTouch Verio Flex^®^ de LifeScan, Milpitas, CA), y se les indicó la necesidad de calibrar el dispositivo MCG al menos tres veces al día (antes de las comidas y al acostarse). También se les pidió que llevaran un registro sencillo de su actividad, ingesta dietética y horas de sueño y vigilia. El dispositivo MCG se retiró el séptimo día tras el ayuno nocturno, y se volvieron a extraer muestras de sangre para determinar la glucosa en ayunas y la HbA_1c_.

De cada participante, se excluyeron del análisis todas las lecturas de MCG de un día determinado si (a) la diferencia absoluta relativa media entre las lecturas de sangre capilar de ese día y los valores de MCG correspondientes era superior al 18 %, y (b) el registro de MCG del día no estaba completo (288 lecturas entre las 0:00 h y las 24:00 h). Los participantes con menos de dos registros diarios completos fueron excluidos del estudio. La glucemia media en 24 horas (24 h-GM) se calculó como la media de las 288 lecturas del día, la SD en 24 horas como la desviación estándar de estas 288 lecturas, el CV en 24 horas como (SD en 24 horas)/(24 h-GM), y la MAGE (amplitud media de las excursiones glucémicas) según lo descrito por Hill et al. [24].

### Análisis bioquímicos

La glucosa se determinó en muestras séricas de participantes en ayunas mediante el método de la glucosa oxidasa-peroxidasa. Los triglicéridos, HDL, LDL, colesterol total y biomarcadores de la función hepática y renal se determinaron mediante métodos enzimáticos en un analizador totalmente automático (Advia 2400 de Siemens Healthcare Diagnostics, Barcelona, España). La glucemia capilar se determinó con glucómetros (OneTouch^®^ Verio^®^ Pro de LifeSpan). La HbA_1c_ se analizó mediante cromatografía líquida de alta resolución en un analizador Menarini Diagnostics HA-8160; todos los valores de HbA_1c_ se convirtieron a valores alineados con el *Diabetes Control and Complications Trial* (DCCT) [25]. De acuerdo al criterio de la ADA [23] se consideraron valores normales de glucosa concentraciones inferiores a 100 mg/dL y concentraciones de HbA_1c_ inferiores a 5.7 %. Todos los análisis de laboratorio se realizaron el mismo día de la recogida de muestras en el Laboratorio de Bioquímica Clínica del Complejo Hospitalario Universitario de Santiago de Compostela (España).

### Análisis estadístico

Se comprobó la normalidad de todas las variables. Los datos sobre variables continuas se resumen como medias ± SD o como medianas con rangos intercuartílicos entre paréntesis. Se calcularon estadísticas descriptivas para todo el grupo de estudio. La significación estadística de las diferencias entre grupos se estimó mediante la prueba t de Student para variables con distribución normal y con la prueba U de Mann-Whitney para variables con distribución no paramétrica. Las correlaciones por pares entre variables se calcularon mediante la r de Pearson o la rho de Spearman. El valor P de 2 colas con un nivel de significación α se fijó en 0,05. Todos los análisis estadísticos se realizaron con SPSS 27 (SPSS, Chicago, IL).

### Declaración ética

El presente estudio fue aprobado por el Comité Ético de Investigación Clínica de Galicia, España (CEIC# 2012–025 y CEIC# 2016–240). Se obtuvo el consentimiento informado por escrito de todos los sujetos, de conformidad con la Declaración de Helsinki.

## Resultados

**Figura 1:**
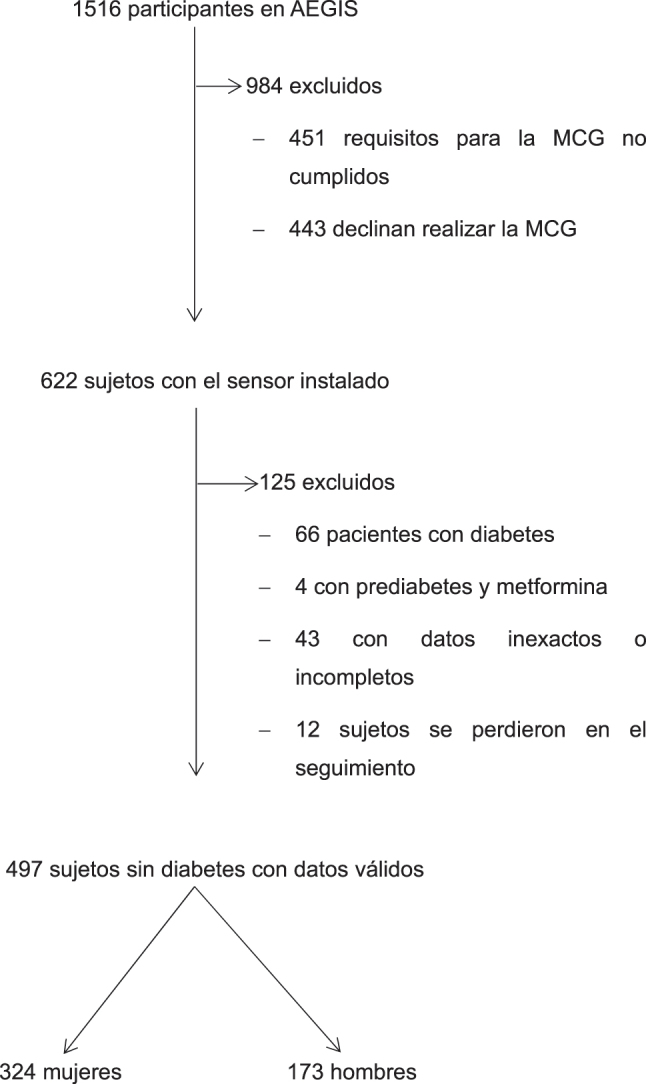
Selección de pacientes.

De los 622 participantes evaluados en este estudio, 497 fueron seleccionados como aptos para su participación (Figura 1). Cuarenta y tres sujetos fueron excluidos debido a datos incompletos o inexactos, o por dificultades en el manejo del sistema. Posteriormente, se excluyeron 70 sujetos: 66 por tener un diagnóstico de diabetes y 4 por estar en tratamiento con metformina para la prediabetes. Finalmente, se perdió el seguimiento de 12 participantes (58 % hombres), estos en promedio eran más jóvenes (37 ± 18 años) y con un IMC de 26,4 ± 4,3 kg/m^2^. Ninguno de ellos presentaba síndrome metabólico. La glucosa en ayunas y la HbA_1c_ fueron, respectivamente, de 88 ± 10 mg/dL y 5,3 ± 0,3 %.

Los participantes proporcionan un total de 2347 días completos de datos de MCG: un 80 % de los participantes aportaron datos de 5 días, un 14,3 % de 4 días, un 3,4 % de 3 días y un 2,3 % de 2 días. De los 497 participantes, 324 (65,2 %) eran mujeres y 173 (34,8 %) hombres. Comparados con el grupo de mujeres (Tabla 1), los hombres presentan concentraciones significativamente más elevadas de IMC, presión sistólica y diastólica, triglicéridos, LDL-colesterol y glucosa en ayunas (p<0,05), y concentraciones de HDL-colesterol significativamente más bajos (p<0,001). No se encontraron diferencias significativas entre hombres y mujeres, en las medidas basales de control glucémico (HbA_1c_), las medidas de variabilidad glucémica proporcionadas por la MCG (SD, CV y MAGE), ni en la glucemia promedio indicada por el sensor.

**Tabla 1:** Características basales de los participantes.

	Total	Hombres	Mujeres	Valor p^a^
Número de participantes	497	173	324	
Edad, años	46,6 ± 13,9	46,1 ± 14,1	46,9 ± 13,8	0,537
Tabaquismo activo	112 (22,5)	47 (27,2)	65 (20,1)	0,071
IMC, kg/m^2^	27,8 ± 5,1	28,8 ± 5,0	27,2 ± 5,0	0,01
Obesidad (IMC ≥30 kg/m^2^)	153 (30,8)	67 (38,7)	86 (26,5)	0,005
Presión sistólica, mmHg	125 ± 15	130 ± 13	123 ± 15	<0,001
Presión diastólica, mmHg	77 ± 8	80 ± 8	75 ± 8	<0,001
eGFR, mL/min/1,73m^2^	112,3 ± 24,6	111,0 ± 23,5	113,0 ± 25,2	0,381
Colesterol total, mg/dL	199 ± 36	198 ± 36	199 ± 35	0,777
Triglicéridos, mg/dL	93 (66–127)	105 (73–151)	87 (65–116)	<0,001
HDL colesterol, mmol/L	61 ± 17	52 ± 14	65 ± 17	<0,001
LDL colesterol, mmol/L	117 ± 30	121 ± 32	115 ± 29	0,023
HbA_1c_, %	5,4 ± 0,3	5,4 ± 0,3	5,3 ± 0,3	0,317
HbA_1c_, mmol/mol	35,0 ± 3,6	35,2 ± 3,4	34,9 ± 3,4	0,317
Glucosa en ayunas, mg/dL	87 ± 11	90 ± 11	86 ± 10	0,002
Parámetros MCG				
Glucosa media, mg/dL	105 ± 8	106 ± 8	105 ± 8	0,151
SD, mg/dL	13,5 ± 4,4	13,2 ± 4,3	13,7 ± 4,3	0,269
CV, %	14,6 ± 4,3	14,4 ± 4,1	14,7 ± 4,4	0,435
MAGE, mg/dL	27,9 ± 9,4	27,9 ± 10,1	27,9 ± 9,0	0,957

Los datos son medias ± SD, medianas [IQR] o [%]. MCG, monitorización continua de glucosa; CV, coeficiente de variación; eGFR, tasa de filtración glomerular estimada; MAGE, amplitud media de las excursiones glucémicas. ^a^Para diferencias entre hombres y mujeres.

Los sujetos sin diabetes fueron seguidos durante una media de 6 años (rango intercuartílico de 4,9 a 7,3 años). El período de seguimiento fue ligeramente superior (p=0,021) en el caso de las mujeres (6,1 (5,0–7,3) años) en comparación con los hombres (5,5 (4,5–7,1) años). Sin embargo, progresaron a diabetes 16 mujeres (4,9 %) y 9 hombres (5,2 %), sin diferencias significativas entre ambos grupos. Inicialmente, las concentraciones de HbA_1c_ y glucosa en ayunas de aquellos que finalmente desarrollaron diabetes fueron significativamente más elevados que los de quienes no progresaron a diabetes (5,8 % vs. 5,3 %; y 102 vs. 86 mg/dL; p<0,001 en ambos casos) (Tabla 2). Comparados con los que no progresaron a diabetes, los participantes que sí desarrollaron diabetes presentaron una variabilidad glucémica significativamente mayor (SD media 18 vs. 13 mg/dL, p<0,001; CV 17 % vs. 14 %, p=0,004; MAGE 36 vs. 27 mg/dL, p<0,001) así como una glucemia media más elevada (115 vs. 105 mg/dL, p<0,001).

**Tabla 2:** Marcadores de control glucémico y de variabilidad glucémica entre sujetos que progresan o no a diabetes.

	Progresan a diabetes de tipo 2		
Variable	No (n=472)	Si (n=25)	Valor p^a^
HbA_1c_,	5,3 (0,3)	5,8 (0,4)	<0,001
HbA_1c_, mmol/mol	35 (3)	40 (4)	<0,001
Glucosa en ayunas, mg/dL	86 (10)	102 (12)	<0,001
Glucosa media, mg/dL	105 (8)	115 (9)	<0,001
SD, mg/dL	13,3 (4,2)	18,4 (5,0)	<0,001
CV, %	14,4 (4,2)	16,9 (4,4)	0,004
MAGE, mg/dL	27,4 (9,2)	35,9 (10,3)	<0,001

Los datos son medias ± SD o medianas [IQR]. CV, coeficiente de variación; MAGE, amplitud media de las excursiones glucémicas. ^a^Para la diferencia entre sujetos que desarrollan y no desarrollan diabetes de tipo 2.

Se realizaron curvas ROC para comparar las AUC (area under the curve) de diferentes parámetros de variabilidad glucémica de la MCG, con el fin de evaluar su capacidad de predicción de la diabetes tipo 2, así como la sensibilidad y especificidad de estas variables. El análisis ROC mostró que la SD tiene la AUC más alta, tanto en la población total (0,81) como en hombres (0,87) y mujeres (0,77) (Tabla 3). En la población sin diabetes, un punto de corte de 14,9 mg/dL para la SD mostró una sensibilidad del 80 % y una especificidad del 72 %. Al comparar hombres y mujeres para la predicción de diabetes, todas las AUC fueron más elevadas en hombres, tanto para la SD (0,87 vs. 0,77), como para el CV (0,74 vs. 0,62) y el MAGE (0,82 vs. 0,72). De las medidas de variabilidad estudiadas, la mayor sensibilidad la tuvo la SD en hombres (88,9 %) y la mayor especificidad la presentó el MAGE en mujeres (78,2 %).

**Tabla 3:** Sensibilidad, especificidad y área bajo la curva para la predicción de diabetes de diferentes parámetros de variabilidad glucémica.

Variable	AUC (95 % CI)	Valor p	Punto de corte	Sensibilidad^a^	Especificidad^a^
Total (n=497)					
SD, mg/dL	0,81 (0,73–0,88)	<0,0001	14,9	80,0	72,3
CV	0,67 (0,56–0,77)	0,001	14,7	68,0	59,8
MAGE, mg/dL	0,76 (0,67–0,85)	<0,001	34,2	58,0	81,5
Hombres (n=173)					
SD, mg/dL	0,87 (0,78–0,96)	<0,001	14,9	88,9	73,0
CV	0,74 (0,61–0,87)	<0,001	14,7	88,7	59,5
MAGE, mg/dL	0,82 (0,70–0,94)	<0,001	28,1	88,8	60,7
Mujeres (n=324)					
SD, mg/dL	0,77 (0,66–0,88)	<0,001	15,4	75,0	74,6
CV	0,62 (0,48–0,76)	0,086	15,5	56,0	66,8
MAGE, mg/dL	0,72 (0,60–0,84)	<0,01	32,3	62,5	78,2

AUC, area under the curve; CV, coeficiente de variación; MAGE, amplitud media de las excursiones glucémicas. ^a^Los datos se informan como porcentajes.

## Discusión

En este estudio prospectivo, que incluye una amplia muestra de participantes sin diabetes representativos de la población general, se han analizado diversos parámetros de variabilidad glucémica obtenidos mediante monitorización continua de glucosa (MCG) como predictores de la progresión a diabetes tipo 2 durante un seguimiento medio de 6 años. Aunque las diferentes métricas estudiadas (SD, CV y MAGE) predicen el desarrollo de diabetes, el análisis ROC indica que la SD tiene la AUC más alta. Además, se observa que, para todos los parámetros estudiados, las AUC son mayores en hombres que en mujeres.

Actualmente, la variabilidad glucémica se destaca como una herramienta valiosa en la evaluación de la gestión de la diabetes, demostrando que es un factor predictivo de las complicaciones de la diabetes. Asimismo, una variabilidad glucémica elevada representa un desafío importante para alcanzar los objetivos de los parámetros tradicionales de control glucémico, como la HbA_1c_. Se sabe que la amplitud de la variabilidad glucémica se correlaciona positivamente con el riesgo de desarrollo de todas las complicaciones crónicas de la diabetes: neuropatía, retinopatía, enfermedades renales crónicas y problemas macrovasculares. Esta asociación está mediada principalmente por el aumento de la inflamación y el estrés oxidativo, que son consecuencia de una mayor variabilidad glucémica [26], 27]. Sin embargo, hasta donde sabemos, no existen estudios prospectivos que evalúen parámetros de la variabilidad glucémica de la MCG como indicadores de la progresión a la diabetes en muestras poblacionales.

El hecho de que los participantes de nuestro estudio con mayor variabilidad glucémica desarrollen más casos de diabetes puede estar parcialmente asociado con una mayor prevalencia de situaciones que favorecen su desarrollo en estos individuos. Los valores de glucosa en sangre son variables homeostáticas con un alto grado de inestabilidad, incluso en periodos cortos de tiempo, y se ha indicado [28] que esta mayor inestabilidad puede estar influida por diversas condiciones fisiológicas (p.ej., ingesta de glucosa, estrés emocional o ejercicio) o patológicas (p.ej., inflamación, infecciones o trastornos endocrinos).

Aunque las guías actuales recomiendan un CV inferior al 36 %, esto es aplicable a pacientes con diabetes, de tal forma que un CV por debajo de 36 % se asocia con una glucemia estable [29]. Sin embargo, en la población sin diabetes, encontramos que la SD tiene una AUC más alta y una mejor sensibilidad que el CV para predecir qué participantes progresan a diabetes. Esto podría deberse a que, a diferencia de los pacientes con diabetes, las oscilaciones en las concentraciones medias de glucemia son mucho menores en individuos sin diabetes. En este estudio, también hemos encontrado que el MAGE, aunque menos sensible, presenta una mayor especificidad para detectar a los participantes que progresan a diabetes, muchos de los cuales pueden tener irregularidades en los hábitos alimenticios. Es importante tener en cuenta que el índice MAGE no permite evaluar la estabilidad de los valores glucémicos ni el tiempo de permanencia en hipoglucemia o hiperglucemia; sin embargo, está diseñado principalmente para proporcionar información sobre la medida en que se producen excursiones glucémicas entre la hipoglucemia en ayunas y la hiperglucemia posprandial [30].

En conclusión, en este estudio prospectivo con aproximadamente 500 participantes sin diabetes representativos de la población general seguidos durante un promedio de 6 años, se muestra que los participantes que progresan a diabetes tipo 2 presentan inicialmente una elevada variabilidad glucémica estimada a través de diferentes métricas de la MCG (SD, CV y MAGE) en comparación con aquellos que no progresan a diabetes. Estos datos proporcionan información fundamental para considerar futuros tratamientos destinados a prevenir el inicio de la diabetes.
